# Clinical implications in the shift of syndecan-1 expression from the cell membrane to the cytoplasm in bladder cancer

**DOI:** 10.1186/1471-2407-14-86

**Published:** 2014-02-13

**Authors:** Makito Miyake, Adrienne Lawton, Yunfeng Dai, Myron Chang, Lourdes Mengual, Antonio Alcaraz, Steve Goodison, Charles J Rosser

**Affiliations:** 1 Cancer Research Institute, Orlando Health, Orlando, FL 32827, USA; 2 Department of Pathology, Orlando Health, Orlando, FL 32806, USA; 3 Department of Biostatistics, The University of Florida, Gainesville, FL 32610, USA; 4 Laboratory and Department of Urology, Hospital Clínic, Universitat de Barcelona, Barcelona, Spain; 5 Nonagen Bioscience Corp, Orlando, FL 32827, USA; 6 Department of Health Sciences Research, Mayo Clinic, Jacksonville, FL 32224, USA; 7 University of Hawaii Cancer Center, Clinical and Translational Research Program, 701 Ilalo Street, Honolulu, HI 96813, USA

**Keywords:** Syndecan, Bladder, Cancer biomarker, Specificity

## Abstract

**Background:**

To determine the diagnostic and prognostic capability of urinary and tumoral syndecan-1 (SDC-1) levels in patients with cancer of the urinary bladder.

**Methods:**

SDC-1 levels were quantitated by enzyme-linked immunosorbent assay (ELISA) in 308 subjects (102 cancer subjects and 206 non-cancer subjects) to assess its diagnostic capabilities in voided urine. The performance of SDC-1 was evaluated using the area under the curve of a receiver operating characteristic curve. In addition, immunohistochemical (IHC) staining assessed SDC-1 protein expression in 193 bladder specimens (185 cancer subjects and 8 non-cancer subjects). Outcomes were correlated to SDC-1 levels.

**Results:**

Mean urinary levels of SDC-1 did not differ between the cancer subjects and the non-cancer subjects, however, the mean urinary levels of SDC-1 were reduced in high-grade compared to low-grade disease (*p* < 0.0001), and in muscle invasive bladder cancer (MIBC) compared to non-muscle invasive bladder cancer (NMIBC) (*p* = 0.005). Correspondingly, preliminary data note a shift from a membranous cellular localization of SDC-1 in normal tissue, low-grade tumors and NMIBC, to a distinctly cytoplasmic localization in high-grade tumors and MIBC was observed in tissue specimens.

**Conclusion:**

Alone urinary SDC-1 may not be a diagnostic biomarker for bladder cancer, but its urinary levels and cellular localization were associated with the differentiation status of patients with bladder tumors. Further studies are warranted to define the potential role for SDC-1 in bladder cancer progression.

## Background

Syndecan 1 (SDC-1) is one of four members of a transmembrane heparan sulfate proteoglycan family. SDC-1 is the major syndecan expressed in epithelia, and it plays a critical role in cellular processes including differentiation, cell adhesion, migration and invasion, and angiogenesis [1-3]. Functions have been ascribed to the extracellular domain that carries glycosaminoglycan (GAG) side chains, to the transmembrane domain and to the cytoplasmic domain that transduces signals from extracellular ligand binding [3]. Altered SDC-1 expression has been reported in a number of malignant tumor types and has been associated with differentiation status and survival [4-6].

Aaboe *et al*., identified SDC-1 as a bladder cancer (BCa) biomarker using gene expression profiling [7]. Through proteomic analyses of voided urines from BCa patients, SDC-1 has also been identified as a potential diagnostic biomarker [8]. However, in a subsequent multiplex biomarker study of 127 subjects, urinary SDC-1 protein could not be confirmed to be significantly elevated in patients with BCa [9]. The observed inconsistency as a diagnostic biomarker may be related to the study cohorts employed to date, but it may also be due in part to the transmembrane nature of SDC-1. Release of SDC-1 into the soluble fraction of the urine is dependent on a number of factors including: cellular turnover, aberrant processing in disease states, release by inflammation-associated shedding [10], and a shift of expression from epithelial to stromal cells in tumors [11].

Herein, we report further evaluation of the potential utility of SDC-1 as a diagnostic and prognostic biomarker in BCa by analysis of a large diverse test cohort through enzyme-linked immunosorbent assay (ELISA), and the investigation of SDC-1 protein expression patterns in bladder tumors through immunohistochemical (IHC) analysis of archival tissue specimens.

## Methods

### Urinary SDC-1 levels

After Institutional Review Board approval by MD Anderson Cancer Center Orlando and Hospital Clínic of Barcelona and written informed consent, voided urines were collected into institutional tissue banks. From these tissue banks in the Departments of Urology from Orlando Health and Hospital Clínic of Barcelona, 308 voided urine samples and associated clinical data were identified. The study cohort consisted of 206 adult subjects with no active BCa or previous history of BCa (47 with voiding symptoms, 44 with urolithiasis, 9 with gross hematuria, 14 with urinary tract infection and 92 without any of the above diagnoses) and 102 subjects diagnosed with *de novo* urothelial carcinoma. Median follow-up of the patients with BCa was 14 months. In our cancer group and hematuria group, imaging of the upper urinary tract and cystoscopy were performed. Furthermore, the histologic subtype, urothelial carcinoma, was confirmed by histological examination of excised tissue in the cancer group.

Voided urine samples were centrifuged to separate the supernatant from the cellular pellet. The supernatant was decanted and aliquoted, and the urinary pellet was snap frozen. Both the supernatant and pellet were stored at -80°C prior to analysis. Urine supernatant protein concentration was determined using Pierce 660-nm Protein Assay Kit (Thermo Fisher Scientific Inc., Waltham, MA, USA). The level of human SDC-1 (Cat# ab46507 Abcam, Cambridge, MA, USA) was monitored in urine samples using a commercial ELISA assay. The assay was conducted according to the manufacturer’s instructions. A calibration curve was prepared using purified standards for SDC-1. Curve fitting was accomplished by either linear or four-parameter logistic regression following manufacturer’s instructions. Laboratory personnel were blinded to final diagnosis.

### Syndecan-1 expression in human bladder tumors

Under Institutional Review Board approval with a waiver of consent, 185 bladder tumor paraffin blocks and eight benign bladder paraffin blocks dating from 2002-2009 were identified in the pathologic archives of Orlando Health Department of Pathology. The eight benign bladder paraffin blocks were from autopsy cases in which there was no record of BCa, hematuria or tobacco use. Median follow-up of the patients was 18 months. All paraffin blocks were examined by H&E for histological verification of urothelial carcinoma only histology. Paraffin blocks were cut 5 μm sections and placed on a Superfrost Plus Microslide. Sections were deparaffinized followed by antigen retrieval using citric acid buffer (pH 6.0, 95°C for 20 min). Slides were treated with 1% hydrogen peroxide in methanol to block endogenous peroxidase activity. After 20 min blocking in phosphate buffered saline (PBS) containing 1% bovine serum albumin (BSA), slides were incubated overnight at 4°C with anti-human SDC-1 antibody (mouse monoclonal–Abcam ab34164, dilution 1/400 in PBS containing 1% BSA). Next, slides were incubated with 2 μg/mL of biotinylated anti-mouse IgG secondary antibody (Vector Laboratories, Burlingame, CA) for 30 min at room temperature. Subsequently, the sections were stained using Standard Ultra-Sensitive ABC Peroxidase Staining kit (Pierce/Thermo Fisher Scientific, San Jose, CA) and 3, 3′-diaminobenzidine (DAB; Vector Laboratories), counterstained by hematoxylin, dehydrated, and mounted with a cover slide. Human liver tissue, known to stain strongly for SDC-1, was used as a positive control and omitting the primary antibody served as the negative control. The above immunostaining for SDC-1 as well as the interpretation of the immunostaining for SDC-1 were based on a previous report by Mukunyadzi, *et al*. [12]. Briefly, the location of immunoreactivity (*e.g.*, nuclear, cytoplasm, cell membrane, and stroma) was noted. The sections were analyzed and staining assessed using a semiquantitative grading system as follows: negative (-), complete lack of staining or staining in <10% of tumor cells; weak (+), staining in 10 to 20% of tumor cells; mild (++), staining in 20 to 50% of tumor cells; moderate (+++), staining in 50 to 70% of tumor cells and; strong (++++), staining in >70% of tumor cells. Using light microscopy, two investigators (MM and AL), who were both blinded to patients’ data, interpreted immunostaining results. A third investigator (CJR) reviewed discrepancies and rendered a final score.

### Data analysis

The Wilcoxon rank sum test was used to determine the association between urinary SDC-1 and BCa. Nonparametric receiver operating characteristic (ROC) curves were plotted and the ability of the urinary SDC-1 biomarker to indicate BCa was estimated by calculating the area under the ROC curves (AUC). The sensitivity and specificity of the biomarker at the optimal cutoff value was defined by calculating the Youden index [13]. The agreement between interpreting SDC-1 immunohistochemistry results between the two investigators was analyzed using kappa statistics with the strength of agreement 0.81-1.00 interpreted as almost perfect. The results are presented as weighted kappa with 95% confidence interval (CI). Comparison of immunohistochemical distribution data was performed using Chi square test. Disease-specific survival (DSS) curves were obtained using the Kaplan-Meier method, and compared by the log rank test for each prognostic variable [14]. Multivariate analysis was performed to identify independent prognostic variables using a stepwise Cox proportional hazards regression model. Statistical significance in this study was set at *p* < 0.05 and all reported *p* values were 2-sided. All analyses were performed using SAS software version 5.00 (San Diego, CA).

## Results

### Urinary SDC-1 ELISA

**Figure 1:**
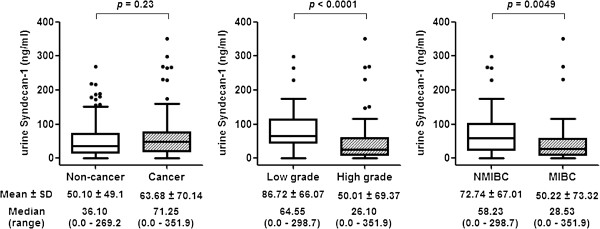
**Urinary Syndecan-1 levels.** Comparison of urinary concentrations of SDC-1 between the cancer and non-cancer groups. In the box-and-whisker plot of urinary concentration of SDC-1, the central box represents the value from the lower to upper quartile. Significance (*p < 0.05*) was assessed by the Wilcoxon rank sum test.

Characteristics of the study cohort of 308 subjects (102 subjects with active BCa and 206 subjects with no evidence of active BCa or a history of BCa) are presented in the Table 1. The median urinary concentration of SDC-1 was not significantly higher overall in subjects with BCa compared to subjects without BCa (71.25 ng/ml *vs*. 36.10 ng/ml, *p* = 0.23) (Figure 1). Neither did SDC-1 levels differ among the groups that made up the diverse control cohort (*p* = 0.562, data not shown). However, a difference in urinary SDC-1 level was noted between patients with tumors of differing grade and invasive subtype. Specifically, low-grade bladder tumors were noted to have higher median urinary SDC-1 levels compared to high-grade bladder tumors (64.55 ng/ml *vs*. 26.1 ng/ml, *p* < 0.0001), and non-muscle invasive bladder cancer (NMIBC) had higher median urinary SDC-1 levels compared to muscle invasive bladder cancer (MIBC) (58.23 ng/ml *vs*. 28.53 ng/ml, *p* = 0.0049) (Figure 1).

**Table 1:** Demographic and clinicopathologic characteristics of 308 subjects comprising ELISA study cohort and 193 subjects comprising IHC study cohort

	**ELISA Cohort**		**IHC Cohort**	
	**BCa (%) n = 102**	**Controls (%) n = 206**	**BCa (%) n = 185**	**Controls (%) n = 8**
Median Age (range, y)	69 (20–93)	56 (18–89)	73 (30–94)	26 (21–43)
Male: Female ratio	84 : 18	152 : 54	143 : 42	4 : 4
Race				
White	91 (89%)	135 (66%)	156 (84%)	N/A
African American	5 (5%)	20 (10%)	8 (4%)	N/A
Other	6 (6%)	51 (24%)	19 (12%)	N/A
Positive FISH	40 / 74 (54%)	2/22 (9%)	N/A	N/A
Suspicious/positive cytology	37 / 94 (39%)	2/22 (9%)	N/A	N/A
Median follow-up (months)	14	4	18	N/A
Clinical stage				
Tis	6 (6%)		17 (9%)	
Ta	41 (40%)		45 (24%)	
T1	14 (14%)		63 (34%)	
≥T2	41 (40%)		60 (33%)	
Tumor grade				
Low	38 (37%)		27 (15%)	
High	64 (63%)		158 (85%)	
Median tumor size (cm)	3.0		3.0	

*IHC* immunohistochemistry, *BCa* bladder cancer, *N/A* not available.

### Immunohistochemical staining of bladder tissue specimens

**Figure 2:**
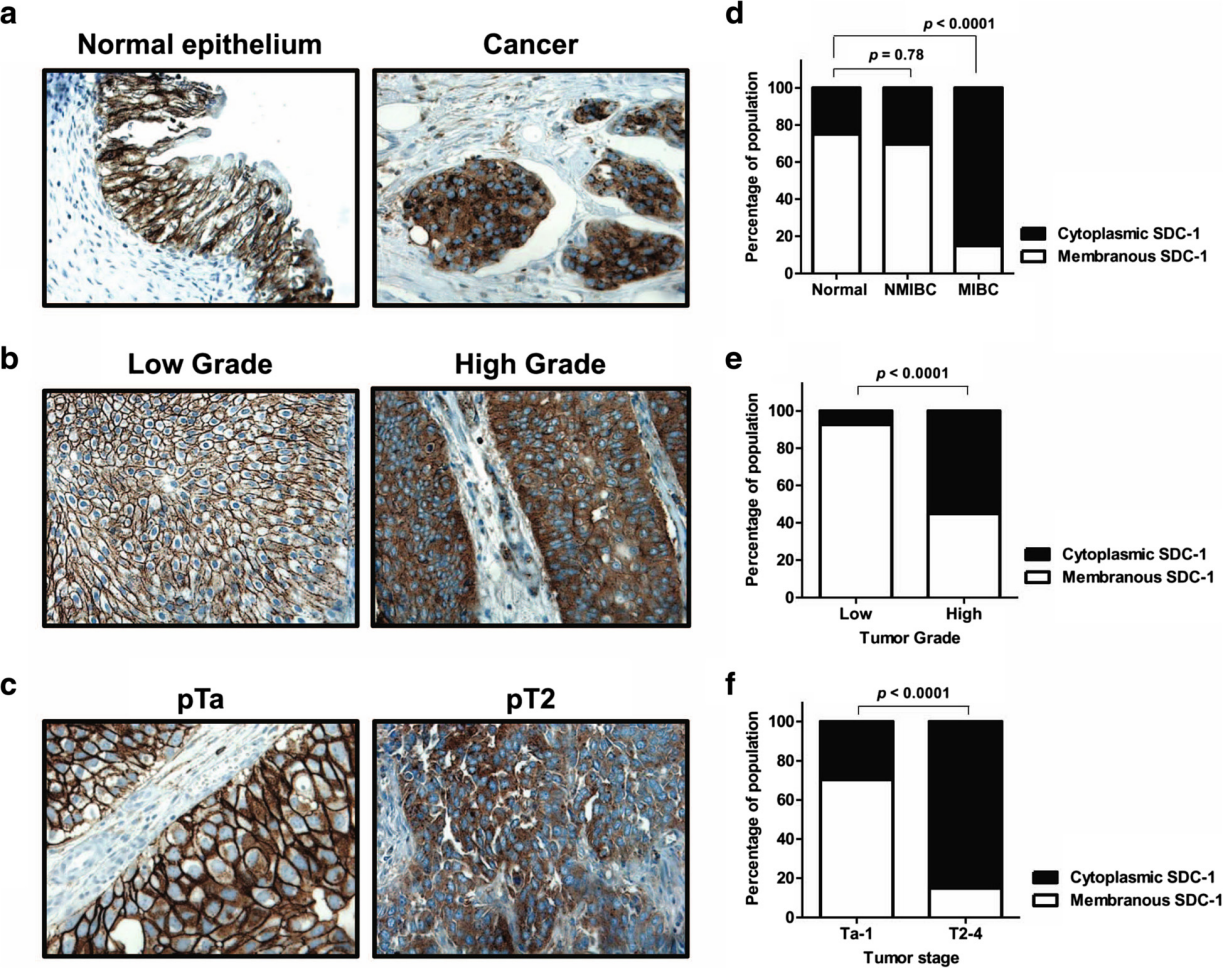
**Expression of Syndecan-1 protein in human bladder tissue. a)** Representative staining of benign bladder epithelium (left) and cancerous bladder (right) showing membranous staining of epithelial cells. **b)** Representative staining of low-grade bladder cancer (left) and high-grade bladder cancer (right). High-grade cancers were noted to have cytoplasmic staining while losing their membranous staining. **c)** Representative staining of low pathologic stage (pTa) bladder cancer (left) and high pathologic stage (pT2) bladder cancer (right). All images were captured at 400× magnification. Column bar graphs illustrate the population of subjects with SDC-1 membrane staining and SDC-1 cytoplasmic staining in **(d)** benign bladder epithelium *vs*. non-muscle invasive bladder cancer (NMIBC) *vs*. muscle invasive bladder cancer (MIBC), **(e)** low-grade tumor *vs*. high-grade tumor and **(f)**, Ta-1 tumor *vs*. T2-4 tumor.

Characteristics of the study cohort of 193 subjects (185 subjects with urothelia carcinoma histology only and 8 subjects with benign bladder histology) are presented in the Table 1. The pathologists’ intra-observer agreement on SDC-1 interpretation and scoring was ‘good’ with a noted kappa score of 0.64 (0.8–1.0, excellent; 0.6–0.8, good; 0.4–0.6, moderate; 0–0.4, poor), 95% CI 0.61–0.68. The percentage agreement was 82.0%. In normal tissue, as well as low-grade disease and NMIBC, over 70% of SDC-1 immunostaining was located within the cellular membrane (Figure 2a) and was graded as moderate (+++) to strong (++++). Minimal immunoreactivity was noted in the stroma. Within bladder tumors, 55% of high-grade tumors (compared to low-grade tumors, *p* < 0.0001) were noted to have increased cytoplasmic expression and reduced membranous expression of SDC-1 (Figure 2b). In the same way, higher stage tumors (T2-4 *vs*. Ta-1, *p* < 0.0001) were noted to have increased cytoplasmic expression and reduced membranous expression of SDC-1 (Figure 2c). Though the location of staining changed from membranous to cytoplasmic amongst high-grade and high stage tumors, immunostaining grading, weak (+) to strong (++++), did not change, illustrating a shift of the ubiquitously expressed SDC-1 from the cellular membrane in well-differentiated, low stage tumors to the cytoplasm in poorly-differentiated, higher stage tumors.

### Analyses of prognostic parameters associated with disease specific survival

**Figure 3:**
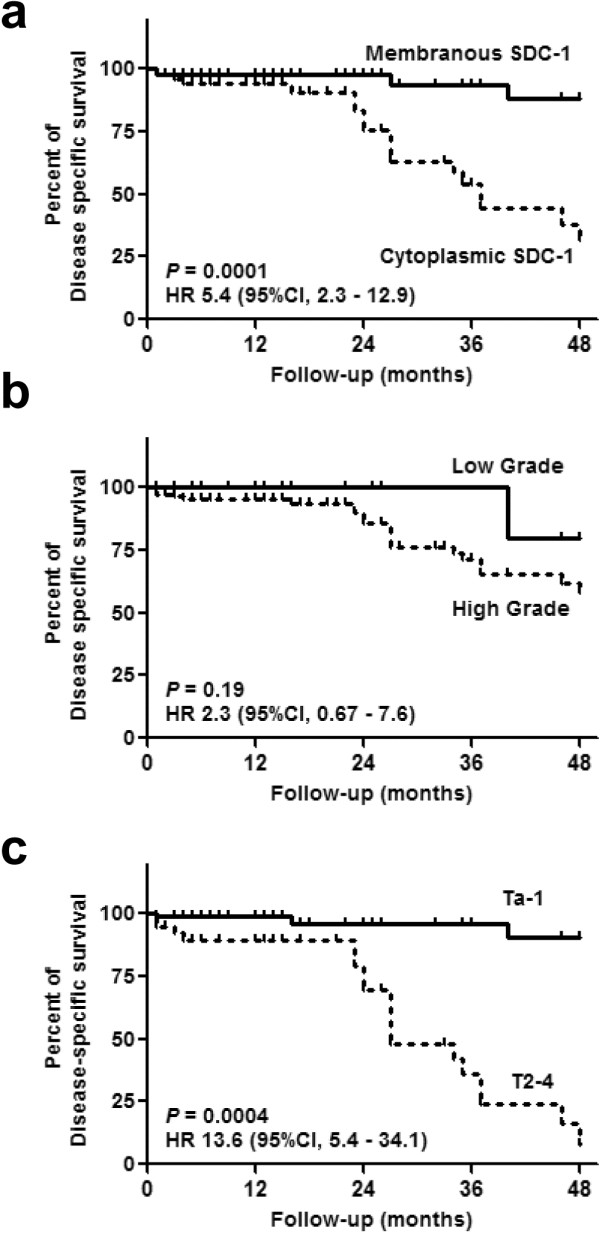
**Kaplan-Meier curves for disease-specific survival.** Disease-specific survival stratified by **(a)** membranous *vs*. cytoplasmic SDC-1, **(b)** low-grade *vs*. high-grade and **(c)** non-muscle invasive bladder cancer (NMIBC) *vs*. muscle invasive bladder cancer (MIBC). HR, hazard ratio; 95% CI, 95% confidence interval.

Univariate analysis revealed that NMIBC and membranous immnostaining for SDC-1 represent favourable prognostic factors associated with disease-specific survival (DSS) (*p* < 0.0001 and *p* = 0.0004, respectively) (Figure 3). However on multivariate analysis (Table 2), only MIBC (hazard ratio [HR] = 21.1, 95% confidence interval [CI] = 4.24–105.1, *p* = 0.0001) proved to be an independent risk factor for DSS, resulting in a significant reduction in survival. Furthermore, MIBC was associated with a significant reduction in overall survival (HR = 9.60, CI: 2.59-35,5, *p* = 0.001).

**Table 2:** Multivariate analysis of disease specific survival and overall survival

**Variables**			**Disease-specific survival**			**Overall survival**		
		**N**	**HR**	**95% CI**	***p***	**HR**	**95% CI**	***p***
Stage								
	NMIBC	125	1			1		
	MIBC	60	21.10	4.24–105.1	0.0001	9.60	2.59–35.5	0.001
SDC-1 expression								
	Membrane	96	1			1		
	Cytoplasm	89	0.87	0.21–3.60	0.85	0.99	0.27–3.60	0.99

*HR* Hazard ratio, *95% CI* 95% confidence interval, *NMIBC* Non-muscle invasive bladder cancer, *MIBC* Muscle invasive bladder cancer.

## Discussion

SDC-1 is expressed mainly in epithelial tissues, hence, studies aiming to address its role in malignancies have focused on carcinoma. In a number of malignancies, the expression of SDC-1 correlates with tumor stage and grade [15-18], but the association between SDC-1 status and BCa has not been extensively studied. Other investigators have reported a positive correlation of SDC-1 with fibroblast growth factors (FGFRs) in bladder tumors, these factors are thought to be key molecules in low-grade BCa [19]. Only Shimada *et al*., have investigated the biologic role of SDC-1 in human BCa cells. In their study, the BCa cell lines, UMUC2 and UMUC3 had SDC-1 expression silenced by siRNA transfection, which led to an induction of apoptosis *in vitro* and a reduction in mouse orthotopic bladder tumor growth [20].

To our knowledge, our study is the largest study to date to evaluate SDC-1 in human bladder tumors both in voided urine and in tumor sections. We used two complimentary approaches to classify SDC-1 expression in human bladder tumors. First, urinary SDC-1 levels were monitored by ELISA in a cohort of 308 subjects. While there was no difference in urinary SDC-1 levels between BCa-bearing subjects and non-BCa bearing subjects (*p* = 0.23), lower urinary levels of SDC-1 were associated with the presence of high-grade tumors and/or MIBC. The prognostic capability of SDC-1 in predicting higher grade and higher stage disease prior to patients undergoing cystoscopy and transurethral resection of bladder tumor has the potential to improve patients’ outcomes. Second, we determined the expression pattern of SDC-1 protein in a cohort of 193 bladder tissue specimens. Though a difference in SDC-1 expression pattern was not seen between bladder tumors and benign bladder histology, possibly due to the small sample size of the benign cohort, a significant shift in cellular localization of SDC-1 was associated with high-grade tumors and MIBC. These tumors tended to lose the distinct membranous staining observed in normal urothelia. The two complimentary approaches utilized in the current study yielded similar inferences, *i.e.*, more aggressive or more advance BCa has less membrane bound SDC-1. If less membrane bound SDC-1 is present in a tumor mass, then it might be expected that less shed or released SDC-1 would be present in the soluble fraction of voided urine from patients with more aggressive or advanced BCa.

Shifts in SDC-1 expression patterns have been alluded to in previous reports, but none in BCa. A study by Mennerich *et al*., described a shift of SDC-1 expression from the epithelial component to the stromal component in solid tumors [11]. An observed overall increase in tumor SDC-1 mRNA was demonstrated by *in situ* hybridization and protein levels confirmed by immunohistochemistry in tumor-associated stromal cells in breast, lung and colon carcinoma. We did not observe this phenomenon in our study, the majority of SDC-1 expression was in the epithelial component of the bladder tumors. The expression pattern shift that our analyses revealed was from distinctly membranous to diffusely cytoplasmic in high-grade and high-stage bladder tumors. This association with disease progression suggests that the loss of SDC-1 function at the cell-surface or cell membrane and thus may facilitate cancer progression and the development of invasive and metastatic disease. Several studies have shown the involvement of cell-surface SDC-1 in cell-cell and cell-matrix adhesion, possibly through the regulation of integrin activities [21]. The loss of SDC-1 at the cell surface by extracellular cleavage can decrease the strength of tumor cell adhesion within the tissue architecture, resulting in an increase in cellular motility. This in turn may allow cancer cells to cross the basement membrane and invade surrounding tissues as well as distant sites [11]. The loss of SDC-1 at the cell-surface could also occur through a switch to translation of alternative, non-membranous isoforms, or by aberrant processing in an advanced tumor. This concept exists for the well-known tumor suppressor gene E-cadherin. Similar to SDC-1, cell-surface E-cadherin assists in cell adhesion and loss of E-cadherin is associated with more aggressive BCa that possess a greater potential to invade and metastasize [22,23]. Though the present studies are quite intriguing, they only elude to a biologic phenomenon which now must be further explored to a) report associated cellular and molecular changes, b) confirm the ELISA and immunohistochemistry results in a large cohort, c) determine which domain (cytoplasmic, transmembrane or extracellular) is shed in voided urine and d) determine in addition to changes in location in expression if there are changes in the quantity of SDC-1 expression between the various disease states. Furthermore, the preliminary nature of our immunohistochemical results should be confirmed in a larger cohort.

## Conclusions

In summary, decreased urinary levels of SDC-1 in BCa patients were associated with high-grade or high-stage disease, and this phenomenon correlated with a shift of SDC-1 protein cellular localization from the cellular membrane to the cytoplasm in these high-grade and high stage bladder tumors. On univariate analysis, loss of membranous localization of SDC-1 was associated with a significant reduction in DSS. This is the first report to describe specific SDC-1 expression changes as being associated with more aggressive, lethal BCa. Further studies are underway to understand the role of SDC-1 in BCa and to investigate the prognostic potential of SDC-1 monitoring in human bladder tumors.

## Competing interests

Dr Goodison and Charles J. Rosser have a competing interest in that they are officers of Nonagen Bioscience Corp, a small biotech company with an interest to develop urinary biomarkers. No other authors possess a competing interest.

## Authors’ contributions

All authors have read and approved the final manuscript. MM, AL: acquisition of data. YD, MC: statistical analysis. LM, AA: clinical samples, drafting of manuscript. SG: study concept and design, drafting of manuscript. CJR: study concept and design, drafting of manuscript, funding.

## Pre-publication history

The pre-publication history for this paper can be accessed here:

http://www.biomedcentral.com/1471-2407/14/86/prepub
